# Assessing Interventions on Crowdsourcing Platforms to Nudge Patients for Engagement Behaviors in Primary Care Settings: Randomized Controlled Trial

**DOI:** 10.2196/41431

**Published:** 2023-07-13

**Authors:** Kay-Yut Chen, Yan Lang, Yuan Zhou, Ludmila Kosmari, Kathryn Daniel, Ayse Gurses, Yan Xiao

**Affiliations:** 1 College of Business University of Texas at Arlington Arlington, TX United States; 2 Department of Business State University of New York at Oneonta Oneonta, NY United States; 3 Department of Industrial, Manufacturing, and Systems Engineering University of Texas at Arlington Arlington, TX United States; 4 College of Nursing and Health Innovation University of Texas at Arlington Arlington, TX United States; 5 Armstrong Institute Center for Health Care Human Factors Anesthesiology and Critical Care, Emergency Medicine, and Health Sciences Informatics, School of Medicine Johns Hopkins University Baltimore, MD United States

**Keywords:** Amazon Mechanical Turk, behavioral interventions, crowdsourcing, medication safety, Mturk, patient engagement, primary care

## Abstract

**Background:**

Engaging patients in health behaviors is critical for better outcomes, yet many patient partnership behaviors are not widely adopted. Behavioral economics–based interventions offer potential solutions, but it is challenging to assess the time and cost needed for different options. Crowdsourcing platforms can efficiently and rapidly assess the efficacy of such interventions, but it is unclear if web-based participants respond to simulated incentives in the same way as they would to actual incentives.

**Objective:**

The goals of this study were (1) to assess the feasibility of using crowdsourced surveys to evaluate behavioral economics interventions for patient partnerships by examining whether web-based participants responded to simulated incentives in the same way they would have responded to actual incentives, and (2) to assess the impact of 2 behavioral economics–based intervention designs, psychological rewards and loss of framing, on simulated medication reconciliation behaviors in a simulated primary care setting.

**Methods:**

We conducted a randomized controlled trial using a between-subject design on a crowdsourcing platform (Amazon Mechanical Turk) to evaluate the effectiveness of behavioral interventions designed to improve medication adherence in primary care visits. The study included a control group that represented the participants’ baseline behavior and 3 simulated interventions, namely monetary compensation, a status effect as a psychological reward, and a loss frame as a modification of the status effect. Participants’ willingness to bring medicines to a primary care visit was measured on a 5-point Likert scale. A reverse-coding question was included to ensure response intentionality.

**Results:**

A total of 569 study participants were recruited. There were 132 in the baseline group, 187 in the monetary compensation group, 149 in the psychological reward group, and 101 in the loss frame group. All 3 nudge interventions increased participants’ willingness to bring medicines significantly when compared to the baseline scenario. The monetary compensation intervention caused an increase of 17.51% (*P*<.001), psychological rewards on status increased willingness by 11.85% (*P*<.001), and a loss frame on psychological rewards increased willingness by 24.35% (*P*<.001). Responses to the reverse-coding question were consistent with the willingness questions.

**Conclusions:**

In primary care, bringing medications to office visits is a frequently advocated patient partnership behavior that is nonetheless not widely adopted. Crowdsourcing platforms such as Amazon Mechanical Turk support efforts to efficiently and rapidly reach large groups of individuals to assess the efficacy of behavioral interventions. We found that crowdsourced survey-based experiments with simulated incentives can produce valid simulated behavioral responses. The use of psychological status design, particularly with a loss framing approach, can effectively enhance patient engagement in primary care. These results support the use of crowdsourcing platforms to augment and complement traditional approaches to learning about behavioral economics for patient engagement.

## Introduction

Research in behavioral economics has suggested principles to influence people’s behaviors, or “nudging” [1-3]. In community settings, especially when compared with hospital settings, health outcomes rely on effective partnerships [4] among health care professionals, patients, and families and “coproduction” of health care work [5]. The application of nudging may provide needed support to encourage patients and families to participate in decisions and management of chronic conditions. Real incentives, such as money or status, are shown to have a clear and universally understood impact. One barrier is the challenge to assess different designs to nudge behaviors. Implementing intervention experiments in community and primary care settings can be exceedingly complex, time-consuming, and expensive. Crowdsourcing could potentially be used to narrow and refine the field of design choices in a timely and cost-efficient manner. For example, large numbers of individuals can be asked to respond to simulated nudging interventions in simulated health care encounters in crowdsourcing experiments. Simulated health care encounters and nudging interventions are akin to role-playing scenarios where the participants are asked to put themselves in, and respond to, simulated versus real scenarios. Although crowdsourcing gains use in health care [6], it is unclear whether participants would respond to simulated incentives in role-playing scenarios. For example, if the nudging choice is to provide financial rewards, no actual rewards are provided in crowdsourcing experiments. Rather, the rewards are simulated in health care scenarios.

This study’s aim was to assess the feasibility of using crowdsourced survey experiments to assess the efficacy of behavioral economics–based interventions in improving patient partnership behavior in their own care, with a particular focus on comparing simulated incentives to actual incentives. Additionally, the study intended to assess the impact of 2 behavioral economics–based intervention designs, psychological rewards and loss framing, on simulated patient partnership behaviors in a simulated primary care setting. Should crowdsourcing be proven feasible, we may use the method to evaluate a large range of design options quickly and efficiently and identify a small subset of candidate interventions for subsequent detailed, multimethod evaluations and implementation trials that can be more strategic and efficient.

We selected bringing medications to primary care visits as the targeted partnership behavior to improve medication safety. Inappropriate medication use in community settings is frequent, as indicated by medication harm related visits to the emergency department [7]. Obtaining accurate medication histories is recommended as a best practice in medication safety, especially in patients with multiple prescribers [8]. Although seemingly logical and straightforward, ensuring medication history accuracy for older adults with multiple comorbid conditions and their related prescriptions is much more complex [9]. Bringing medications to primary care visits is advocated as a way for patients to be involved in improving the accuracy of medication histories [10]. In addition, care professionals may be able to use medication bottles to engage patients in discussions and to have a comprehensive understanding of all of the drugs that patients take. Despite efforts, many patients do not bring their medications to their clinic visit [11]. Behavioral economics based nudging options may be effective in improving patient’s partnership behavior.

## Methods

### Overview

This study was part of a project aiming to understand how to improve medication safety in primary care clinic settings. We conducted a survey-based experiment on the impact of simulated incentives on willingness to engage in a targeted behavior that supports medication review: bringing medications to the clinic at each visit. We used a crowdsourcing platform, Amazon Mechanical Turk (MTurk), to conduct the experiment. The experiment used “role-playing” methodologies in marketing science [12,13] and neuroscience [14-16] to study the impact of different nudging strategies in simulated scenarios. The targeted behavior in the experiment was bringing medicines to a primary care visit.

The study was designed to evaluate whether web-based crowdsourcing surveys could be used to assess the effectiveness of interventions based on behavioral economic principles. Feasibility was measured by examining whether web-based participants responded to simulated incentives in the same way they would have responded to actual incentives. Specifically, we examined whether the treatment effect for simulated incentives was in the same direction and statistically significant as the treatment effect for actual incentives. This approach allowed us to determine the feasibility of using simulated incentives as a proxy for actual incentives in behavioral economics–based interventions. The study also aimed to provide insights into the impact of interventions by using role-playing scenarios, which were tested on MTurk.

### Behavioral Economics–Based Intervention Design

We designed 3 nudging scenarios to test 1 type of simulated physical incentive and 2 types of simulated psychological incentives based on the principles of behavioral economics (Table 1).

1. Monetary compensation: the targeted behavior is rewarded financially. It is well known that money is a strong motivator [17]. We implemented an intervention that relied on simulated incentives to assess its comparable effect to that of actual monetary incentives.
2. Status effect: the targeted behavior is rewarded with a status as psychological incentive, which can be impactful and is more affordable than physical incentives as an effective motivator [18].
3. Loss frame: the absence of the target behavior is penalized by removing a status. In comparison with gaining a status as an incentive, the loss frame (penalty to be paid if the desired behavior is absent) has been shown to have a strong effect [3].

**Table 1:** The baseline scenario and 3 nudging scenarios to simulate behavioral interventions.

Please envision yourself in the following situation	
Baseline scenario	You are taking 8 different medicines prescribed by 3 doctors. On Monday you have an appointment for a follow-up visit with your family doctor. You plan to take buses, which on a good day will take about 45 minutes. The doctor’s office always asks you to bring all medicines with you.
Simulated monetary compensation scenario	Baseline scenario plus If you bring all your medicines, you will receive $15.
Simulated status effect scenario	Baseline scenario plus If you bring all your medicines, you will achieve VIP (very involved patient) status. You will receive a certificate for the VIP status.
Simulated loss frame scenario	Baseline scenario plus You have VIP (very involved patient) status in this clinic. If you forget to bring all your medicines, you will lose this status.

### Crowdsourcing Experimental Design

We conducted a randomized controlled trial using a between-subject design in which each participant was given either a baseline scenario or 1 of the 3 nudging scenarios (Table 1). The scenarios differed only in the design of the specific behavioral intervention. Participants were asked to read the description of the scenario and then answer survey questions to assess the impact of simulated incentives on the targeted behavior of bringing medicines to clinic visits. The survey questions were carefully reviewed by our research team to ensure that they effectively measured the intended constructs. It is important to note that those questions were designed to assess participants’ stated intentions rather than their actual behavior.

### Ethics Approval

This study received ethical approval from the University of Texas at Arlington’s institutional review board prior to survey administration (2022-0026). It involves no more than minimal risk to the participants. Informed consent was obtained from all participants prior to their participation in the study. Participants were informed of the study purpose, their rights as research participants, and the nature of the data that would be collected. To protect the privacy and confidentiality of the study participants, all study data were anonymized and deidentified. The compensation for participation was set at US $0.35 on MTurk.

### Eligibility, Consent, and Recruitment

To attain a representative sample, we limited our participants’ geographic location to the United States. They were adults aged 18 years or older and had a positive reputation on MTurk (had completed more than 100 tasks with a minimum 95% approval rating) [19,20]. We used the SoPHIE (Software Platform for Human Interaction Experiments) software system to administer the surveys. Participants were screened for eligibility using SoPHIE and were asked to read and sign a consent form before completing the surveys. We also administered a brief demographic survey to collect information about participants’ age, race, ethnicity, gender identity, education, income level, and number of chronic diseases at the end of the study. The recruitment period was completed in under 2 weeks, and we did not encounter missing data as this study was conducted through SoPHIE through MTurk, which strictly enforces data completeness.

### Measurement and Analysis

We measured willingness to bring medicines with 4 survey questions on 5-point Likert scales (Table S1 in Multimedia Appendix 1), with a higher score indicating a greater willingness to bring medicine to the clinic. We aggregated the responses to the 4 questions with an average (“willingness score”), following a common practice [21]. We included an additional reverse-coded question (Table S1 in Multimedia Appendix 1) to assess if participants’ answers were intentional [22]. We used Cronbach α to assess the internal consistency of the survey questions and to determine if any participants may have answered randomly.

We aggregated the responses and used the Wilcoxon rank sum test and mean score test to assess the impact of different nudging interventions on willingness scores. We further assessed if demographics (age, household income, education, and race) and chronic medical conditions influenced the impact of nudging.

We also combined several demographic variables for ease of analysis. Age (in years) was combined into 3 groups: younger than 35 years, between 35 and 49 years, and equal to and older than 50 years. Education level was combined into 3 groups: less than a bachelor’s degree, bachelor’s degree, and a graduate degree. Race was combined into 2 groups: White and non-White. Income (in US $) was combined into 3 groups: less than US $40,000, US $40,000-$80,000, and greater than US $80,000. The number of chronic diseases was combined into 2 groups: 0 and more than 0.

## Results

### Overview

A total of 569 study participants were recruited, with 132 in baseline, 187 in monetary compensation, 149 in status effect, and 101 in loss frame scenarios (self-reported demographic information in Table 2). Table 3 summarizes the summary statistics of the scores. We also report the Cronbach alpha [23] of each measure that consists of more than one question (Table S4 in Multimedia Appendix 1).

**Table 2:** MTurk participant demographic information (N=569).

Characteristics			Values
Age (years), mean (SD)			40.25 (12.92)
**Sex, n (%)**			
	Male	279 (49.03)	
	Female	286 (50.26)	
	Other	4 (0.70)	
**Highest education level, n (%)**			
	Less than a bachelor’s degree	151 (26.54)	
	Bachelor’s degree	282 (49.56)	
	Graduate degree	136 (23.90)	
**Ethnicity, n (%)**			
	Hispanic	101 (17.75)	
	Non-Hispanic	468 (82.25)	
**Race, n (%)**			
	Non-White	99 (17.40)	
	White	470 (82.60)	
**Household income (US $), n (%)**			
	Under 40,000	192 (33.75)	
	40,000-80,000	264 (46.40)	
	Above 80,000	113 (19.86)	
**Number of chronic medical conditions, n (%)**			
	0	240 (42.18)	
	>0	303 (53.24)	
	N/A^a^	26 (4.57)	

^a^N/A: not available.

**Table 3:** Summary statistics of survey questions, by scenario.

Scenarios	Baseline, mean (SD)	US $15 incentive, mean (SD)	Psychological incentive, mean (SD)	Psychological incentive with loss frame, mean (SD)
Willingness Score	3.59 (0.94)	4.22 (0.83)	4.02 (0.91)	4.47 (0.70)
Reverse Coded Score	3.15 (1.33)	2.28 (1.44)	2.10 (1.27)	1.94 (1.33)

### Experimental Results

#### Result 1

Both monetary and psychological incentives increased willingness scores.

All 3 interventions increased participants’ willingness to bring medicines significantly when compared to the baseline scenario. The monetary incentive caused an increase of 17.51% (*P*<.001), psychological rewards on status effect increased willingness by 11.85% (*P*<.001), and a loss frame on the psychological rewards increased willingness by 24.35% (*P*<.001).

#### Result 2

Psychological incentive with or without a loss frame has a different impact on willingness scores compared to monetary incentive.

The willingness score for the psychological incentive was 4.82% lower (*P*=.047) than the monetary compensation. Conversely, the willingness score for the psychological incentive in the loss frame was 5.82% higher (*P*=.008) than the monetary compensation.

#### Result 3

Responses to the reverse-coding question were consistent with the willingness questions.

In the reverse-coding questions, all 3 interventions decreased participants’ willingness to bring medicines significantly when compared to the baseline scenario. The monetary compensation intervention caused a decrease of 27.71% (*P*<.001), psychological rewards on status effect decreased willingness by 33.34% (*P*<.001), and a loss frame on the psychological rewards decreased willingness by 38.42% (*P*<.001).

#### Result 4

Our investigation examined a range of demographic factors, including age (younger than 35 years, between 35 and 49 years, and 50 years or older), education level (less than a bachelor’s degree, bachelor’s degree, or a graduate degree), race (White vs non-White), income level (less than US $40,000, between US $40,000 and US $80,000, and over US $80,000), and the presence of chronic conditions, to determine if they had any impact on the effectiveness of the interventions implemented. However, our results, as displayed in Table S2 in Multimedia Appendix 1 did not reveal any consistent evidence of such an impact.

We have also incorporated additional information regarding the demographic characteristics of the MTurk participants who were included in each scenario. Specifically, we have included distribution graphs that provide a visual representation of these demographic factors, which can be found in Table S5 in Multimedia Appendix 1.

## Discussion

### Feasibility of Using Simulated Incentives to Nudge Behaviors

We found that crowdsourced surveys with simulated monetary incentives resulted in expected simulated behavioral responses as in actual incentives with the same direction, demonstrating the feasibility of using crowdsourced surveys to evaluate behavioral economics–based interventions. A consistency check showed that study participants understood the simulated nudging options and responded in expected manners, suggesting that this approach may be a viable and efficient method for evaluating interventions in health care. A simulated psychological status design, particularly with a loss framing design, had a statistically significant (*P*<.001) impact on the targeted behavior and thus should be considered as an effective behavioral intervention design in primary care to enhance patient engagement. These results support the use of crowdsourcing platforms in efficiently and rapidly reaching large numbers of individuals to assess the efficacy of behavioral interventions, which can augment and complement traditional intervention design and evaluation approaches.

### Main Study Results

We concluded that both simulated monetary and psychological incentives had positive impacts on participants’ willingness to bring their medications during a simulated visit to the primary care clinic. The simulated monetary incentive at the level of US $15 was stronger than the psychological incentive.

We also found strong evidence that the simulated psychological incentive could be strengthened by the inclusion of the loss frame. In addition, results were consistent between the willingness scores and the reverse-coded scores. Thus, we conclude that participants understood the questions and their answers to simulated incentives were valid and not the result of a random process.

### Crowdsourcing

Previous uses of crowdsourcing in health-related research include soliciting research priorities and preferences in back pain [24], image classification in ophthalmology [25], informing the design and implementation of HIV and sexual health interventions [26], improving the quality and speed of cancer research [27], and effect of physician gender and race [28]. This study provided initial evidence of using crowdsourcing in designing behavioral economics–inspired nudging options in engaging patients through simulated incentives.

Implementing interventions to engage patients in complex and high-tempo clinical care settings requires significant planning and effort. Any intervention introduced to a complex sociotechnical work system, especially without adequate proactive analysis, may have unintended consequences and actually reduce the system’s overall performance rather than improving it. For interventions that focus explicitly on “behavior modification,” there can be numerous design options, which make it infeasible to implement within the actual clinical work and evaluate. In this study, we found crowdsourcing platform–based analysis of behavioral intervention designs to be feasible, useful in providing valid data, and flexible enough to be complementary to other well-known intervention design and implementation approaches that may require significant time and resources.

Web-based crowdsourcing platforms have become increasingly popular for connecting workers to on-demand tasks. Literature suggests that behavioral experiments conducted on MTurk are as valid as those conducted with more traditional methods in laboratories, such as in a study comparing supply chain experiments conducted on MTurk with those in laboratories [29]. On MTurk, there are roughly 226,000 workers available in the United States. Of these, approximately 80,000 have been active in recent years [30]. In addition, approximately 40,000-50,000 new workers sign up each year. Other platforms include Survey Monkey [31] and Qualtrics [32].

### Comparative Advantages of Crowdsourcing Methods for Design

Compared to traditional methods of collecting behavioral data, such as field studies in a clinical setting, interviews, and focus groups, crowdsourcing is more efficient, less expensive, and easier to implement. This efficiency is driven by 2 factors. First, crowdsourcing platforms provide access to a large participant pool around the clock at affordable prices. In this study, data gathering on each scenario took only hours to accomplish, versus typically days or weeks with traditional methods. Second, the platform eliminates the need to plan the logistics and training of protocol personnel, a must for methods such as field studies.

Further, we use a scenario-based framework, common in marketing studies, to design survey experiments with simulated incentives. That is, we use descriptive language to create the scenarios in participants’ minds and solicit their responses when they put themselves in this “thought experiment.” This technique sidesteps the limitations of creating interventions in a physical environment (eg, training of clinical staff, creating testing materials, etc). There are no limits to the scenarios we can create with simulated incentives. This flexibility not only allows us to test promising designs, but also enables us to gather data on risky and impractical designs to further our understanding and explore the design space more effectively.

### Crowdsourced Data Validity

By using simulated encounters and simulated nudging options, experiment data from crowdsourcing might not reflect the impact of nudge options in the real world. The study participants may not reflect the targeted patient population [24]. We used a presurvey to filter out participants that were not in the targeted population, but such filtering was limited and relied on respondents’ own responses. The targeted nudging behaviors were general and less context dependent (eg, responding to gain-loss framing); a sample from the general participant pool provided by the platform might suffice. Crowdsourcing also lacks direct control and monitoring of how study participants respond. By using surveys in scenario-based simulated experiments, the self-reported propensity of targeted behaviors may be biased. We used a between-subject design, so each participant responded to only 1 scenario. We used multiple items to measure the targeted behavior, along with a reverse-coded item to assess internal consistency (eg, whether the participants provide valid, as opposed to random, answers).

### Limitations

This study’s participant pool on crowdsourcing did not reflect older adults, who have the highest risk for medication harm [7]. Crowdsourcing platforms require a level of technological familiarity that older adults tend to lack, especially those with low socioeconomic status and cognitive declines.

Due to the novelty of our method and the lack of literature support for this specific approach, we did not perform a power calculation for this study. However, we acknowledge that this is a limitation and may have impacted the precision of our estimates and our ability to detect small effects. Instead of conducting a power analysis, we opted to increase our sample size as needed, considering the relatively low cost of our sample.

Crowdsourcing incentives study participants to complete the study as quickly as possible, although the use of multiple items and reverse-coded questions reduced this risk. The recruitment process relied on study participant self-selection, and thus selection bias was not assessed.

The simulated responses in the study may be different from the actual responses in real health care encounters. MTurk users may choose to respond as fast as possible. We instituted measures to counter this risk, such as using reverse-coded questions to screen for noninvestment and filtering patterned responses.

### Conclusions

This study demonstrates the feasibility of crowdsourcing to assess the impact of 3 behavioral interventions, or nudging options, on simulated medication safety partnership behaviors in a simulated primary care encounter. Our experiments targeted bringing medications to office visits. This patient partnership behavior is often advocated but not widely adopted. We tested and observed the expected impact from behavioral economics–based designs to nudge this behavior through psychological rewards and loss framing against a monetary reward design.

Crowdsourcing platforms such as MTurk are efficient in terms of time and cost to assess design options. Thus, they can be useful to augment and complement traditional approaches to designing ways to engage patients in health behaviors. Our findings revealed that crowdsourced surveys with simulated monetary compensation resulted in the expected simulated behavioral responses as actual incentives. We also found that none of the demographic factors (age, education level, race, income level, and the presence of chronic conditions) had a significant impact on the effectiveness of the interventions. We believe that this information is important for health care practitioners and policy makers, as it suggests that the interventions may be equally effective across diverse populations. In addition, this study suggests that a psychological incentive, specifically the status effect, combined with loss framing may be a good candidate for a clinical trial.

From a practice perspective, these results suggest that psychological incentives, specifically the status effect, combined with loss framing may be a good candidate for a clinical trial of behavioral interventions and medication reconciliation.

## Appendix

Multimedia Appendix 1 Survey questions.

Multimedia Appendix 2 CONSORT e-HEALTH Checklist V1.6.2.

## Abbreviations

MTurk: Amazon Mechanical Turk
SoPHIE: software platform for human interaction experiments

## Data Availability

The data sets generated and analyzed during this study are available in Multimedia Appendix 1.
