# Supplementary material for: Assessing Interventions on Crowdsourcing Platforms to Nudge Patients for Engagement Behaviors in Primary Care Settings: Randomized Controlled Trial
Source: J Med Internet Res. 2023 Jul 13;25:e41431. doi: 10.2196/41431 (PMC10375278; doi:10.2196/41431)
Supplement: Multimedia Appendix 1 [file jmir_v25i1e41431_app1.docx]

## Appendix

Table S1. Survey questions.

|  |  |
| --- | --- |
| Q1 | I will definitely bring all my medicines as asked. |
| Q2 | A friend told me that he has lost medicines when bringing them to the clinic. I still will definitely bring all my medicines as asked. |
| Q3 | I will definitely bring all my medicines if the provider reminds me on the phone a day before my appointment. |
| Q4 | I find it easy to bring all my medicines if I wanted to. |
| Q5 | I find it difficult to bring all my medicines to my doctor appointment. |

Table S2. Willingness score compared to baseline by demographic factors.

|  | Monetary Compensation | | | | Status Effect | | | | Loss Frame | | | |
| --- | --- | --- | --- | --- | --- | --- | --- | --- | --- | --- | --- | --- |
| **Age** | Estimate | SE | *P* | n | Estimate | SE | *P* | n | Estimate | SE | *P* | n |
| Intervention group | 0.156 | 0.038 | <.001 | 79 | 0.053 | 0.042 | .202 | 59 | 0.179 | 0.043 | <.001 | 38 |
| Age group 1 (35-49) | 0.011 | 0.042 | .791 | 52 | 0.011 | 0.043 | .798 | 52 | 0.011 | 0.040 | .781 | 52 |
| Age group 2 (≥50) | 0.074 | 0.058 | .202 | 19 | 0.074 | 0.060 | .217 | 19 | 0.074 | 0.055 | .181 | 19 |
| Intervention*Age group 1 | 0.020 | 0.055 | .721 | 73 | 0.063 | 0.062 | .309 | 47 | 0.044 | 0.064 | .488 | 34 |
| Intervention*Age group 2 | -0.053 | 0.073 | .471 | 35 | 0.081 | 0.075 | .283 | 43 | 0.051 | 0.076 | .502 | 29 |
| Baseline Age group 0 (<35) | 0.633 | 0.028 | <.001 | 61 | 0.633 | 0.029 | <.001 | 61 | 0.633 | 0.027 | <.001 | 61 |
| We did not find any evidence to suggest that age has an impact on the willingness to bring medications during the baseline or when compared to nudging interventions. | | | | | | | | | | | | |
|  |  |  |  |  |  |  |  |  |  |  |  |  |
|  | Monetary Compensation | | | | Status Effect | | | | Loss Frame | | | |
| **Education** | Estimate | SE | *P* | n | Estimate | SE | *P* | n | Estimate | SE | *P* | n |
| Intervention group | 0.153 | 0.060 | .012 | 47 | 0.053 | 0.064 | .409 | 53 | 0.105 | 0.062 | .092 | 34 |
| Edu group 1 (Bachelor's degree) | -0.151 | 0.057 | .009 | 79 | -0.151 | 0.061 | .014 | 79 | -0.151 | 0.056 | .007 | 79 |
| Edu group 2 (Graduate degree) | -0.096 | 0.063 | .125 | 36 | -0.096 | 0.067 | .152 | 36 | -0.096 | 0.061 | .118 | 36 |
| Intervention*Edu group 1 | -0.006 | 0.068 | .928 | 100 | 0.055 | 0.075 | .468 | 56 | 0.114 | 0.073 | .120 | 47 |
| Intervention*Edu group 2 | -0.035 | 0.078 | .650 | 40 | -0.002 | 0.082 | .979 | 40 | 0.171 | 0.085 | .046 | 20 |
| Baseline Edu group 0 (less than bachelor's degree) | 0.765 | 0.052 | <.001 | 17 | 0.765 | 0.055 | <.001 | 17 | 0.765 | 0.051 | <.001 | 17 |
| We did not find any consistent evidence to suggest that education has an impact on the willingness to bring medications during the baseline or when compared to nudging interventions. | | | | | | | | | | | | |
|  |  |  |  |  |  |  |  |  |  |  |  |  |
|  | Monetary Compensation | | | | Status Effect | | | | Loss Frame | | | |
| **Race** | Estimate | SE | *P* | n | Estimate | SE | *P* | n | Estimate | SE | *P* | n |
| Intervention group | 0.162 | 0.058 | .005 | 36 | 0.196 | 0.071 | .006 | 19 | 0.262 | 0.064 | <.001 | 20 |
| Race group 1 (White) | 0.051 | 0.050 | .308 | 108 | 0.051 | 0.052 | .334 | 108 | 0.051 | 0.048 | .291 | 108 |
| Intervention*Race group 1 | -0.006 | 0.064 | .931 | 151 | -0.106 | 0.077 | .173 | 130 | -0.053 | 0.071 | .460 | 81 |
| Baseline Race group 0 (non-white) | 0.607 | 0.045 | <.001 | 24 | 0.607 | 0.047 | <.001 | 24 | 0.607 | 0.043 | <.001 | 24 |
| We did not find any evidence to suggest that race has an impact on the willingness to bring medications during the baseline or when compared to nudging interventions. | | | | | | | | | | | | |
|  |  |  |  |  |  |  |  |  |  |  |  |  |
|  | Monetary Compensation | | | | Status Effect | | | | Loss Frame | | | |
| **Income** | Estimate | SE | *P* | n | Estimate | SE | *P* | n | Estimate | SE | *P* | n |
| Intervention group | 0.198 | 0.044 | <.001 | 65 | 0.193 | 0.049 | <.001 | 51 | 0.220 | 0.048 | <.001 | 36 |
| Income group 1 (40 - 80k) | 0.069 | 0.043 | .106 | 78 | 0.069 | 0.045 | .125 | 78 | 0.069 | 0.041 | .090 | 78 |
| Income group 2 (>80k) | 0.055 | 0.068 | .423 | 14 | 0.055 | 0.072 | .446 | 14 | 0.055 | 0.065 | .400 | 14 |
| Intervention *Income group 1 | -0.083 | 0.056 | .139 | 89 | -0.150 | 0.062 | .017 | 65 | -0.056 | 0.065 | .387 | 32 |
| Intervention*Income group 2 | 0.013 | 0.083 | .878 | 33 | -0.074 | 0.088 | .401 | 33 | 0.073 | 0.082 | .375 | 33 |
| Baseline Income group 0 (<40k) | 0.602 | 0.035 | <.001 | 40 | 0.602 | 0.036 | <.001 | 40 | 0.602 | 0.033 | <.001 | 40 |
| We did not find any consistent evidence to suggest that income has an impact on the willingness to bring medications during the baseline or when compared to nudging interventions. | | | | | | | | | | | | |
|  |  |  |  |  |  |  |  |  |  |  |  |  |
|  | Monetary Compensation | | | | Status Effect | | | | Loss Frame | | | |
| **Chronic** | Estimate | SE | *P* | n | Estimate | SE | *P* | n | Estimate | SE | *P* | n |
| Intervention group | 0.196 | 0.038 | <.001 | 77 | 0.130 | 0.043 | .003 | 57 | 0.215 | 0.041 | <.001 | 49 |
| Chronic group 1 (>0) | -0.034 | 0.038 | .368 | 70 | -0.034 | 0.041 | .397 | 70 | -0.034 | 0.037 | .356 | 70 |
| Intervention*Chronic group 1 | -0.064 | 0.050 | .198 | 98 | -0.036 | 0.056 | .520 | 87 | 0.004 | 0.056 | .944 | 48 |
| Baseline Chronic group 0 (=0) | 0.668 | 0.029 | <.001 | 57 | 0.668 | 0.031 | <.001 | 57 | 0.668 | 0.028 | <.001 | 57 |
| We did not find any evidence to suggest that the presence of chronic conditions has an impact on the willingness to bring medications during the baseline or when compared to nudging interventions. | | | | | | | | | | | | |

Table S3. Participants’ average response, by scenario.

|  | Baseline | Monetary Compensation | Status Effect | Loss Frame |
| --- | --- | --- | --- | --- |
|  |  |  |  |  |
| Q1 | 3.52 | 4.37 | 4.01 | 4.59 |
| Q2 | 3.48 | 4.06 | 3.89 | 4.34 |
| Q3 | 3.65 | 4.33 | 4.15 | 4.66 |
| Q4 | 3.72 | 4.13 | 4.03 | 4.28 |
| Q5 | 3.15 | 2.28 | 2.10 | 1.94 |
| Note: Questions are coded with the scale from 1 to 5. A higher score indicates a higher willingness to bring medicine to the clinic, except in the reverse coded question 5 where a lower score indicates a higher willingness to bring medicine. | | | | |

Table S4. Survey questions’ Cronbach's alpha, by scenario

| Scenarios | Baseline | Monetary Compensation | Status Effect | Loss Frame |
| --- | --- | --- | --- | --- |
|  |  |  |  |  |
| Willingness Score | 0.809 | 0.821 | 0.778 | 0.829 |
| Reverse Coded Score | - | - | - | - |

Table S5. MTurk participant demographic information by intervention

| Code Book |  |
| --- | --- |
| Income | 1= "Less than $20,000"; 2= "$21,000 – $30,000"; 3= "$31,000 to $40,000"; 4= "$41,000 to $50,000"; 5= "$51,000 to $60,000"; 6= "$61,000 to $70,000"; 7= "$71,000 to $80,000"; 8= "$Above $80,000" |
| Education | 1 = "lessthanhighschool"; 2 = "highschool"; 3 = "associate"; 4 = "bachelor"; 5 = "master"; 6 = "doctor" |
| Race | 1 = "Native"; 2 = "Asian"; 3 = "Black"; 4 = "Pacific"; 5 = "White" |
| Ethnicity | 0 = "No"; 1 = "Yes" |
| Gender | 0 = Female; 1 = Male |
| Chronic disease | 0= "0"; 1= "1-2"; 2= "3-5"; 3= "6-9"; 4= "more than 9" |

Table S6. Correlation analysis of participants' responses to each question

| Baseline | Q1 |  | Q2 |  | Q3 |  | Q4 |  |
| --- | --- | --- | --- | --- | --- | --- | --- | --- |
|  | Coef. | *P* | Coef. | *P* | Coef. | *P* | Coef. | *P* |
| Q2 | 0.445 | <.001 |  |  |  |  |  |  |
| Q3 | 0.612 | <.001 | 0.448 | <.001 |  |  |  |  |
| Q4 | 0.519 | <.001 | 0.487 | <.001 | 0.572 | <.001 |  |  |
| Q5 | -0.161 | .065 | -0.037 | .673 | -0.119 | .176 | -0.078 | .374 |
|  |  |  |  |  |  |  |  |  |
| Monetary Compensation | Q1 |  | Q2 |  | Q3 |  | Q4 |  |
| $15 | Coef. | *P* | Coef. | *P* | Coef. | *P* | Coef. | *P* |
| Q2 | 0.535 | <.001 |  |  |  |  |  |  |
| Q3 | 0.645 | <.001 | 0.460 | <.001 |  |  |  |  |
| Q4 | 0.598 | <.001 | 0.465 | <.001 | 0.499 | <.001 |  |  |
| Q5 | -0.321 | <.001 | -0.301 | <.001 | -0.314 | <.001 | -0.415 | <.001 |
|  |  |  |  |  |  |  |  |  |
| Status Effect | Q1 |  | Q2 |  | Q3 |  | Q4 |  |
|  | Coef. | *P* | Coef. | *P* | Coef. | *P* | Coef. | *P* |
| Q2 | 0.516 | <.001 |  |  |  |  |  |  |
| Q3 | 0.527 | <.001 | 0.511 | <.001 |  |  |  |  |
| Q4 | 0.517 | <.001 | 0.368 | <.001 | 0.360 | <.001 |  |  |
| Q5 | -0.425 | <.001 | -0.261 | .001 | -0.179 | .029 | -0.604 | <.001 |
|  |  |  |  |  |  |  |  |  |
| Loss Frame | Q1 |  | Q2 |  | Q3 |  | Q4 |  |
|  | Coef. | *P* | Coef. | *P* | Coef. | *P* | Coef. | *P* |
| Q2 | 0.566 | <.001 |  |  |  |  |  |  |
| Q3 | 0.645 | <.001 | 0.511 | <.001 |  |  |  |  |
| Q4 | 0.523 | <.001 | 0.564 | <.001 | 0.481 | <.001 |  |  |
| Q5 | -0.213 | .032 | -0.359 | <.001 | -0.265 | .008 | -0.512 | <.001 |
