# Supplementary material for: Assessing Interventions on Crowdsourcing Platforms to Nudge Patients for Engagement Behaviors in Primary Care Settings: Randomized Controlled Trial
Source: J Med Internet Res. 2023 Jul 13;25:e41431. doi: 10.2196/41431 (PMC10375278; doi:10.2196/41431)
Supplement: Multimedia Appendix 2 [file jmir_v25i1e41431_app2.pdf]

CONSORT-EHEALTH Checklist V1.6.2 Report	Manuscript Number	41431

(based on CONSORT-EHEALTH V1.6), available at [http://tinyurl.com/consort-ehealth-v1-6].

Date completed

6/11/2023 22:28:57

by

Ludmila Kosmari

Assessing interventions on crowdsourcing platforms to nudge patients for engagement behaviors in primary care settings: randomized controlled trial

TITLE

1a-i) Identify the mode of delivery in the title

"interventions on crowdsourcing platforms"

1a-ii) Non-web-based components or important co-interventions in title

1a-iii) Primary condition or target group in the title

"patients from primary care"

ABSTRACT

1b-i) Key features/functionalities/components of the intervention and comparator in the METHODS section of the ABSTRACT

"We conducted a randomized controlled trial using a between-subject design on a crowdsourcing platform (Amazon Mechanical Turk) to evaluate the effectiveness of behavioral interventions designed to improve medication adherence in primary care visits"

1b-ii) Level of human involvement in the METHODS section of the ABSTRACT

1b-iii) Open vs. closed, web-based (self-assessment) vs. face-to-face assessments in the METHODS section of the ABSTRACT

1b-iv) RESULTS section in abstract must contain use data

1b-v) CONCLUSIONS/DISCUSSION in abstract for negative trials

INTRODUCTION

2a-i) Problem and the type of system/solution

"Research in behavioral economics has suggested principles to influence people's behaviors, or "nudging" [1-3]". "In community settings, especially when compared with hospital settings, health outcomes rely on effective partnerships [4] among health care professionals, patients, and families and "coproduction" of health care work [5]. The application of nudging may provide needed support to encourage patients and families to participate in decisions and management of chronic conditions. Real incentives, such as money or status, are shown to have a clear and universally understood impact. One barrier is the challenge to assess different designs to nudge behaviors. Implementing intervention experiments in community and primary care settings can be exceedingly complex, time-consuming, and expensive. Crowdsourcing could potentially be used to narrow and refine the field of design choices in a timely and cost-efficient manner".

2a-ii) Scientific background, rationale: What is known about the (type of) system

"Research in behavioral economics has suggested principles to influence people's behaviors, or "nudging". Indirectly, the study is based on the premised of nudging as a technique to influence behavior.

Does your paper address CONSORT subitem 2b?

"Research in behavioral economics has suggested principles to influence people's behaviors, or "nudging".

METHODS

3a) CONSORT: Description of trial design (such as parallel, factorial) including allocation ratio

We conducted a randomized controlled trial using a between-subject design in which each participant was given either a baseline scenario or 1 of the 3 nudging scenarios (Table 1).

3b) CONSORT: Important changes to methods after trial commencement (such as eligibility criteria), with reasons

The methods employed in this study did not undergo any modifications during the study

3b-i) Bug fixes, Downtimes, Content Changes

4a) CONSORT: Eligibility criteria for participants

implicit, participants were all users of Mturk internet platform

4a-i) Computer / Internet literacy

4a-ii) Open vs. closed, web-based vs. face-to-face assessments:

the sample was recruited online exclusively, this was a web-based intervention
"We conducted a survey-based experiment on the impact of simulated incentives on willingness to engage in a targeted behavior that supports medication review: bringing medications to the clinic at each visit. We used a crowdsourcing platform, Amazon Mechanical Turk (MTurk), to conduct the experiment."

4a-iii) Information giving during recruitment

4b) CONSORT: Settings and locations where the data were collected

"We employed the SoPHIE (Software Platform for Human Interaction Experiments) software system to administer the surveys". "To attain a representative sample, we limited our participants' geographic location to the United States".

4b-i) Report if outcomes were (self-)assessed through online questionnaires

"We employed the SoPHIE (Software Platform for Human Interaction Experiments) software system to administer the surveys." "Participants were screened for eligibility using SoPHIE".

4b-ii) Report how institutional affiliations are displayed

5) CONSORT: Describe the interventions for each group with sufficient details to allow replication, including how and when they were actually administered

5-i) Mention names, credential, affiliations of the developers, sponsors, and owners

5-ii) Describe the history/development process

5-iii) Revisions and updating

5-iv) Quality assurance methods

5-v) Ensure replicability by publishing the source code, and/or providing screenshots/screen-capture video, and/or providing flowcharts of the algorithms used

5-vi) Digital preservation

5-vii) Access

"[Participants] were adults aged 18 years or older and had a positive reputation on MTurk (had completed more than 100 tasks with a minimum 95% approval rating"

5-viii) Mode of delivery, features/functionalities/components of the intervention and comparator, and the theoretical framework

"We employed the SoPHIE (Software Platform for Human Interaction Experiments) software system to administer the survey". "A total of 569 study participants were recruited, with 132 in baseline, 187 in monetary compensation, 149 in status effect, and 101 in loss frame scenarios". Although an actual theory is not highlighted, the study purpose conceptually rests on the theoretical premise that "Research in behavioral economics has suggested principles to influence people's behaviors, or "nudging".

5-ix) Describe use parameters

5-x) Clarify the level of human involvement

5-xi) Report any prompts/reminders used

no prompts or reminders were used; the users completed the task in one session upon logging into the survey

**5-xii) Describe any co-interventions (incl. training/support)**		
this item was not applicable		
**6a) CONSORT: Completely defined pre-specified primary and secondary outcome measures, including how and when they were assessed**		
"We used Cronbach alpha to assess the internal consistency of the survey questions and to determine if any participants may have answered randomly."		
**6a-i) Online questionnaires: describe if they were validated for online use and apply CHERRIES items to describe how the questionnaires were designed/deployed**		
**6a-ii) Describe whether and how "use" (including intensity of use/dosage) was defined/measured/monitored**		
**6a-iii) Describe whether, how, and when qualitative feedback from participants was obtained**		
**6b) CONSORT: Any changes to trial outcomes after the trial commenced, with reasons**		
"We employed the SoPHIE (Software Platform for Human Interaction Experiments) software system to administer the surveys". "To attain a representative sample, we limited our participants' geographic location to the United States".		
**7a) CONSORT: How sample size was determined**		
**7a-i) Describe whether and how expected attrition was taken into account when calculating the sample size**		
**7b) CONSORT: When applicable, explanation of any interim analyses and stopping guidelines**		
"We used Cronbach alpha to assess the internal consistency of the survey questions and to determine if any participants may have answered randomly."		
**8a) CONSORT: Method used to generate the random allocation sequence**		
Participant randomization to one of the intervention groups was done by the software		
**8b) CONSORT: Type of randomisation; details of any restriction (such as blocking and block size)**		
no restriction of randomization was identified		
**9) CONSORT: Mechanism used to implement the random allocation sequence (such as sequentially numbered containers), describing any steps taken to conceal the sequence until interventions were assigned**		
allocation concealment is not applicable		
**10) CONSORT: Who generated the random allocation sequence, who enrolled participants, and who assigned participants to interventions**		
randomization was performed by software; none of the investigators were involved in randomization, allocation or sequencing		
**11a) CONSORT: Blinding - If done, who was blinded after assignment to interventions (for example, participants, care providers, those assessing outcomes) and how**		
**11a-i) Specify who was blinded, and who wasn't**		
blinding of participant is not applicable		
**11a-ii) Discuss e.g., whether participants knew which intervention was the "intervention of interest" and which one was the "comparator"**		
**11b) CONSORT: If relevant, description of the similarity of interventions**		
The similarity only pertains to baseline scenario, but were subsequently were randomized as follows: "a total of 569 study participants were recruited, with 132 in baseline, 187 in monetary compensation, 149 in status effect, and 101 in loss frame scenario."		
**12a) CONSORT: Statistical methods used to compare groups for primary and secondary outcomes**		
There was no attrition identified		
**12a-i) Imputation techniques to deal with attrition / missing values**		
There was no attrition identified		
**12b) CONSORT: Methods for additional analyses, such as subgroup analyses and adjusted analyses**		
The demographic subgroup analyses: in addition to age and gender, we assessed education level, race, income and number of chronic illnesses.		
RESULTS		
**13a) CONSORT: For each group, the numbers of participants who were randomly assigned, received intended treatment, and were analysed for the primary outcome**		
"A total of 569 study participants were recruited, with 132 in baseline, 187 in monetary compensation, 149 in status effect, and 101 in loss frame scenario".		
**13b) CONSORT: For each group, losses and exclusions after randomisation, together with reasons**		
There was no attrition identified. Losses and exclusions after randomization are not applicable.		
**13b-i) Attrition diagram**		
**14a) CONSORT: Dates defining the periods of recruitment and follow-up**		
"The recruitment period was completed in under two weeks, and we did not encounter missing data as this study was conducted through SoPHiE through MTurk, which strictly enforces data completeness".		
**14a-i) Indicate if critical "secular events" fell into the study period**		
**14b) CONSORT: Why the trial ended or was stopped (early)**		
"The recruitment period was completed in under two weeks, and we did not encounter missing data as this study was conducted through SoPHiE through MTurk, which strictly enforces data completeness". Data collection stopped at the end of recruitment, upon completion of the survey, as anticipated.		
**15) CONSORT: A table showing baseline demographic and clinical characteristics for each group**		
Table 2 provides necessary data		
**15-i) Report demographics associated with digital divide issues**		
These requirements were fulfilled, see Table 2		
**16a) CONSORT: For each group, number of participants (denominator) included in each analysis and whether the analysis was by original assigned groups**		
**16-i) Report multiple "denominators" and provide definitions**		
This is not applicable due to the nature of the methodology		
**16-ii) Primary analysis should be intent-to-treat**		
**17a) CONSORT: For each primary and secondary outcome, results for each group, and the estimated effect size and its precision (such as 95% confidence interval)**		
Results for outcome are provided in means and standard deviation Table 3		
**17a-i) Presentation of process outcomes such as metrics of use and intensity of use**		
**17b) CONSORT: For binary outcomes, presentation of both absolute and relative effect sizes is recommended**		
this is not applicable, outcomes were not measured as binary variables		
**18) CONSORT: Results of any other analyses performed, including subgroup analyses and adjusted analyses, distinguishing pre-specified from exploratory**		
subgroup/ adjusted analyses are not applicable		
**18-i) Subgroup analysis of comparing only users**		
**19) CONSORT: All important harms or unintended effects in each group**		
No known/ unintended harm to participant occurred		
**19-i) Include privacy breaches, technical problems**		
**19-ii) Include qualitative feedback from participants or observations from staff/researchers**		
DISCUSSION		
**20) CONSORT: Trial limitations, addressing sources of potential bias, imprecision, multiplicity of analyses**		
**20-i) Typical limitations in ehealth trials**		
study limitations were addressed		
**21) CONSORT: Generalisability (external validity, applicability) of the trial findings**		
**21-i) Generalizability to other populations**		
**21-ii) Discuss if there were elements in the RCT that would be different in a routine application setting**		
**22) CONSORT: Interpretation consistent with results, balancing benefits and harms, and considering other relevant evidence**		

**22-i) Restate study questions and summarize the answers suggested by the data, starting with primary outcomes and process outcomes (use)**		
"We found that crowdsourced surveys with simulated monetary incentives resulted in expected simulated behavioral responses as in actual incentives with the same direction, demonstrating the feasibility of using crowdsourced surveys to evaluate behavioral economics-based interventions. A consistency check showed that study participants understood the simulated nudging options and responded in expected manners, suggesting that this approach may be a viable and efficient method for evaluating interventions in health care. A simulated psychological status design, particularly with a loss framing design, had a statistically significant (P<.001) impact on the targeted behavior and thus should be considered as an effective behavioral intervention design in primary care to enhance patient engagement. These results support the use of crowdsourcing platforms in efficiently and rapidly reaching large numbers of individuals to assess the efficacy of behavioral interventions, which can augment and complement traditional intervention design and evaluation approaches"		
**22-ii) Highlight unanswered new questions, suggest future research**		
Other information		
**23) CONSORT: Registration number and name of trial registry**		
this subitem was addressefd		
**24) CONSORT: Where the full trial protocol can be accessed, if available**		
study protocol is available upon request / contact PI		
**25) CONSORT: Sources of funding and other support (such as supply of drugs), role of funders**		
information about funding and support is provided		
**X26-i) Comment on ethics committee approval**		
**x26-ii) Outline informed consent procedures**		
**X26-iii) Safety and security procedures**		
**X27-i) State the relation of the study team towards the system being evaluated**		
